# *In vivo* and *in vitro* study on the combination extract of *Curcuma zedoaria* and Astragalus membranaceus as an adjuvant for PRRSV inactivated vaccine and its preliminary application in piglets

**DOI:** 10.3389/fmicb.2024.1470297

**Published:** 2024-11-22

**Authors:** Teng Tu, Guidong Zhang, Chengchao Du, Yan Luo, Xueping Yao, Zexiao Yang, Meishen Ren, Yin Wang, Dike Jiang

**Affiliations:** ^1^Key Laboratory of Animal Diseases and Human Health of Sichuan Province, Sichuan Agricultural University, Chengdu, China; ^2^College of Veterinary Medicine, Sichuan Agricultural University, Chengdu, China; ^3^Chengdu Agricultural College, Chengdu, China

**Keywords:** porcine reproductive and respiratory syndrome virus, the CZ-AM extract, the optimal ratio, adjuvant, TLR4/NLRP3/IL-1β

## Abstract

**Background:**

Porcine Reproductive and Respiratory Syndrome Virus (PRRSV) is a single-stranded positive-sense RNA virus that severely impacts the global swine industry. Recently, variation and recombination of this pathogen have led to more recombinant strains. However, commercial PRRSV vaccines provide limited protection, and no effective therapeutic drugs are available in clinical settings. Astragalus membranaceus (AM) has anti-inflammatory and immune-enhancing properties, while *Curcuma zedoaria* (CZ) possesses anti-inflammatory, antibacterial, and antitumor effects. The combination of AM and CZ, originating from the “Lichong Tang” recorded in the “Intergrating Chinese and Western Medicine” offers complementary and synergistic benefits.

**Methods:**

In this study, the AM extract, CZ extract, and various ratios of CZ-AM extracts were prepared using the decoction method. The NADC30-like PRRSV strain SCCD22 was used for viral challenge. The optimal ratio and mode of action of the CZ-AM extract on Pams-163 were assessed by measuring viral copies and mRNA expression of cytokines. Subsequently, the optimal ratio of combined extracts identified *in vitro* was used as an adjuvant for the PRRSV inactivated vaccine in 28-day-old piglets. Clinical symptom observations, peripheral blood lymphocyte proliferation assays, levels of PRRSV antibody, cytokine secretion, and NLRP3 inflammasome mRNA were conducted to evaluate the potential of the CZ-AM extract as an adjuvant for the PRRSV inactivated vaccine.

**Results:**

Results showed that the CZ-AM extract inhibited PRRSV *in vitro*, with the best ratio of CZ to AM being 1:4. Animal experiments demonstrated that using the CZ-AM extract as an adjuvant for the PRRSV inactivated vaccine significantly increased the body weight of piglets, elevated serum PRRSV antibody levels, and enhanced the piglets’ inflammatory response. mRNA analysis indicated that the CZ-AM extract inhibited key inflammatory pathways (TLR4/NLRP3/IL-1β), reducing the expression of inflammatory factors. Lymphocyte proliferation assays indicated that the CZ-AM extract significantly stimulated T and B lymphocyte proliferation.

**Conclusion:**

This study not only deepens the understanding of the pharmacological effects of AM and CZ but also expands the application of traditional Chinese medicine in the prevention and control of animal diseases. Furthermore, it provides new insights and methods for optimizing PRRSV vaccines, offering significant scientific value and broad application prospects.

## 1 Introduction

Astragalus membranaceus (AM), also known as “huangqi” in China, is the dried root of the leguminous plants Astragalus membranaceus (Fisch.) Bge. var. mongholicus (Bge.) Hsiao or Astragalus membranaceus (Fisch.) Bge. It is widely distributed in China, particularly in regions such as Beijing, Tianjin, Hebei, Shanxi, and Sichuan. It is harvested in the spring and autumn, with the fibrous roots and root heads removed before drying in the sun. AM possesses tonic, hepatoprotective, diuretic, and expectorant properties (National Pharmacopoeia Commission, 2020) and has been shown to exhibit immunomodulating, antihyperglycemic, antiinflammatory, antioxidant, and antiviral activities, among others (Bedir et al., 2000; Choi et al., 2007; Chan et al., 2009; Wang et al., 2009; Zhu et al., 2009; Li et al., 2010). In traditional Chinese medicine, AM offers numerous benefits, such as enhancing the body’s immune system, treating lung deficiency, and aiding in wound healing (Zhang and Tao, 2016).

*Curcuma zedoaria* (CZ) also known as “ezhu” in China, is the dried rhizome of the Zingiberaceae plants Curcuma phaeocaulis Val., Curcuma kwangsiensis S.G. Lee et C.F. Liang, or Curcuma wenyujin Y.H. Chen et C. Ling (National Pharmacopoeia Commission, 2020). CZ is produced in several regions in China, including Taiwan, Fujian, Jiangxi, Guangdong, Guangxi, Sichuan, and Yunnan. It is also found in India and Malaysia. CZ has a bitter taste, a warm nature, and is non-toxic. According to the ancient Chinese medical text “Compendium of Materia Medica” (Ben Cao Gang Mu), “Curcuma can treat severe chest pain caused by cold air. It is also suitable for individuals with chronic, recurrent chest and abdominal pain.” Modern research has found that the main component of CZ is volatile oil (Zhu and Dai, 2020).

The combination of AM and CZ originates from the traditional Chinese medical formula “Li Chong Tang,” documented by the modern Chinese physician Xichun Zhang in his work “Intergrating Chinese and Western Medicine.” This formula is primarily used to treat Qi (The concept of traditional Chinese medicine, meaning vital energy for life) deficiency and blood stasis. CZ helps to eliminate blood stasis, while AM protects and nourishes the Qi and blood, allowing for the removal of stasis without damaging the vital energy. Furthermore, AM replenishes Qi and blood, and with the aid of CZ, it promotes the circulation of Qi and blood, achieving the effect of supplementation without stagnation, thereby enhancing the vitality. Once the vital energy is robust, it further enhances CZ’s ability to eliminate pathological factors, making the combination highly effective (Wang, 2019). Currently, in traditional Chinese medicine, there are several formulas that utilize the combination of AM and CZ, such as “Qizhu Feixian Decoction” and “Qizhu Antifibrosis Granules.” These formulations have been proven to have good clinical efficacy in combating inflammation and treating lung diseases (Liu et al., 2008; Dai, 2012; Chen et al., 2021).

In recent years, there have been several studies on the combination of AM and CZ. Wu (Wu et al., 2018) measured the effective component content in single decoctions of Astragali Radix, single decoctions of Curcumae Rhizoma, and their combined decoction using high-performance liquid chromatography (HPLC). The results showed that in the single decoction of Astragali Radix, the contents of astragaloside, astragalus saponin I, astragalus saponin II, calycosin, and formononetin were 0.0943, 0.3836, 0.3770, 0.0803, and 0.0349 mg/g, respectively. In the single decoction of Curcumae Rhizoma, the contents of curdione and curcumol were 0.9242 and 0.5230 mg/g, respectively. When combined, the content of these components increased by 2 to 3 times, indicating that the saponins and flavonoids in Astragali Radix have a solubilizing effect on the lipid components in Curcumae Rhizoma.

Yin et al. (2018) used ultra high performance liquid chromatography coupled to triple quadrupole tandem mass spectrometry method (UPLC-QQQ-MS) to measure 17 active components in Astragali Radix and Curcumae Rhizoma. They found that, compared to single decoctions, the combined decoction significantly increased the content of lipophilic components such as curcumenol, curdione, isocurcumenol, furanodienone, curcumol, and germacrone. This indicates that the combination of Astragali Radix and Curcumae Rhizoma can promote the dissolution of active ingredients. Wang (2021) utilized network pharmacology and transcriptomics to investigate the targets and mechanisms by which the combination of AM and CZ can inhibit the growth and metastasis of lung cancer. Her experimental results indicated that the anti-lung cancer mechanism of the CZ-AM combination is related to its regulation of tumor angiogenesis and immune function, thereby impacting the tumor microenvironment.

In this study, we reference the traditional Chinese medicine formula “Qizhu Feixian Decoction” (Liu et al., 2008; Dai, 2012; Chen et al., 2021) and selects a ratio range of CZ to AM from 1:2 to 1:5. Initially, we conducted *in vitro* studies to determine the optimal ratio and interaction mode of CZ-AM extracts on the porcine alveolar macrophages-163 (Pams-163). Subsequently, we used the optimal extract ratio of CZ-AM, identified through *in vitro* screening, as an adjuvant for the PRRSV inactivated vaccine (CH-1a) in 28-day-old piglets. The potential of CZ-AM extracts as an adjuvant was evaluated through clinical symptom observation, peripheral blood lymphocyte proliferation tests, PRRSV antibody levels, and cytokine detection.

This study not only deepens the understanding of the pharmacological effects of CZ-AM, but also expands the application scope of traditional Chinese medicine in the prevention and control of animal diseases. Furthermore, it provides new insights and methods for optimizing PRRSV vaccines, underscoring significant scientific value and broad application prospects.

## 2 Materials and methods

### 2.1 Determination of TCID50 for PRRSV SCCD22

Trypsin Pams-163 (PRRSV receptor CD163 was expressed and transfected into primary alveolar macrophage passage cell line 3D4/21 (CRL-2843). The Pams-163 was maintained and provided by the Animal Quarantine Laboratory of Sichuan Agricultural University) into a 96-well plate. At 80–90% confluency, dilute the PRRSV SCCD22 (GenBank ID: OR670493.1, Lineage 1.8, NADC30-Like strain) in RPMI 1640 medium (2% FBS) in tenfold serial dilutions (10^−1^ to 10^−10^). Add 100 μL of each dilution per well, with eight replicates per dilution, and use 100 μL of RPMI 1640 medium (2% FBS) as a negative control. Incubate at 37°C, 5% CO_2_ for 72–120 h, monitoring daily for CPE. Calculate the TCID50 using the Reed-Muench method.

### 2.2 *In vitro* screening of the ratio of the CZ-AM extract

#### 2.2.1 Preparation of water extracts

Following the method described by Zhu (2019), water decoction was used to separately prepare decoctions of Astragalus slices, Curcuma slices, and a combined decoction of Astragalus and Curcuma slices. The resulting decoctions were then concentrated to form extracts. These extracts were dried to granules, resulting in the AM extract, CZ extract, and CZ-AM extract.

#### 2.2.2 Effects of extracts on Pams-163 using the CCK-8 assay

This study investigated the effects of the AM extract, CZ extract, and different ratios of CZ-AM extracts (with CZ to AM ratios ranging from 1:2 to 1:5) on the viability of Pams-163. The concentrations tested were 1,000 μg/mL, 500 μg/mL, 250 μg/mL, 125 μg/mL, 60 μg/mL, 30 μg/mL, 15 μg/mL, and 7.5 μg/mL. Each concentration was tested in six replicates. A cell control group (cells + RPMI 1640 medium + CCK-8 solution) and a blank control group (RPMI 1640 medium + CCK-8) were also included. After incubating at 37°C for 48 h, 10 μL of CCK-8 reagent was added to each well. The plates were then incubated at 37°C for an hour, and the optical density (OD) was measured at 450 nm using a microplate reader. Cell viability was calculated using the following formula:


$$\begin{array}{l} \mathrm{Cell viability}\;\left(\%\right)=\\ \left(\begin{array}{l} \left(\mathrm{ODexperiment}-\mathrm{ODblank}\right)/\\ \left(\mathrm{ODcontrol}-\mathrm{ODblank}\right) \end{array}\right)\times 100\% \end{array}$$

#### 2.2.3 Screening the mode of action of extracts on PRRSV

Pams-163 were cultured in cell culture plates and cultured until they reached approximately 80% confluence. The following experimental groups were set up: PRRSV infection group, direct effect group (extracts and virus mixed in equal ratios simultaneously), prevention effect group (extracts added before PRRSV), inhibition effect group (PRRSV added before extracts), adsorption effect group (extracts and PRRSV mixed in equal ratios and adsorbed at 4°C), and a blank control group. The specific treatments for each group are as follows:

Virus group: The culture medium was discarded, and 400 μL of PRRSV virus solution (MOI = 0.1) was added to each well.

Direct effect group: Based on the safe concentration of the extracts, the extracts and PRRSV virus solution (MOI = 0.1) were mixed in a 1:1 ratio, and 400 μL of this mixture was added to each well.

Prevention effect group: Based on the safe concentration of the extracts, 400 μL of the extracts were added to each well. After incubating for 4 h, the extract was discarded, and 400 μL of PRRSV virus solution (MOI = 0.1) was added to each well. The plates were incubated at 37°C with 5% CO_2_ for 1.5 h, and then the virus solution was discarded.

Inhibition effect group: Based on the safe concentration of the extracts, each well was inoculated with 400 μL of PRRSV virus solution (MOI = 0.1) and incubated at 37°C with 5% CO2 for 1.5 h. The virus solution was then discarded, and based on the safe concentration of the extracts, 400 μL of the extracts were added to each well.

Adsorption effect group: Based on the safe concentration of the extracts, the extracts and PRRSV virus solution (MOI = 0.1) were mixed in a 1:1 ratio, and 400 μL of this mixture was added to each well. The plates were placed at 4°C for 1.5 h to allow adsorption, and then the virus solution was discarded.

Blank control group: Cells were retained without any treatment.

After these treatments, RPMI 1640 medium (containing 2% FBS) was added to each group, and the plates were incubated at 37°C with 5% CO2. After 24 h of incubation, the total RNA was extracted. The viral copy number was determined using RT-qPCR (Qiu et al., 2020), the primer information (see Supplementary Table S1).

#### 2.2.4 Detection of cytokine relative mRNA expression in PRRSV-infected cells treated with extracts

Using the optimal mode of action and concentration of the extracts identified in previous experiments, we investigated the changes in relative mRNA expression of cytokine in Pams-163 infected with PRRSV. Primers were designed based on sequences available on NCBI using Primer Premier 5 (Table 1). And all primers were synthesized by Tsingke Biotechnology Co., Ltd. (Beijing, China). The relative mRNA expression of each gene were calculated using the 2^−ΔΔCt^ method.

**Table 1:** RT-qPCR primers.

Primer name	The Genbank accession number of reference genes	Sequence (5′-3′)	Length of products (bp)
β-actin-F	XM_021086047.1	CTCCATCATGAAGTGCGACGT	114
β-actin-R		GTGATCTCCTTCTGCATCCTGT	
IL-1β-F	NM_001302388.2	GCCCTGTACCCCAACTGGTA	61
IL-1β-R		CCAGGAAGACGGGCTTTTG	
TNF-α-F	NM_214022.1	CGACTCAGTGCCGAGATCAA	58
TNF-α-R		CCTGCCCAGATTCAGCAAAG	
IFN-α-F	JQ 839262.1	AGACACCTGGTTCATCTCGG	58
IFN-α-R		GGAGGAAGAATGGGCTTGTTAGT	

### 2.3 Determination of Astragaloside IV and Curcuma volatile oil in combined extracts of AM and CZ

The content of the main components (Astragaloside IV and Curcuma volatile oil) in the extracts used in this study was determined according to the methods described in the Chinese Pharmacopoeia (2020) (National Pharmacopoeia Commission, 2020).

Determination of Astragaloside IV (C41H68O14): The extracts were sent to Chengdu Alfa Biotechnology Co., Ltd. (Chengdu, China) for the determination of Astragaloside IV content using high-performance liquid chromatography (HPLC); Determination of Curcuma volatile oil: The content of Curcuma volatile oil in the extracts was determined by the distillation method as specified in the Chinese Pharmacopoeia (2020) (National Pharmacopoeia Commission, 2020).

### 2.4 Preliminary study on the CZ-AM extract as vaccine adjuvants for piglets

The PRRSV inactivated vaccine (CH-1a strain, purchased from JOFUNHWA Biotechnology Co., Ltd.) was used in conjunction with the optimal ratio of AM and CZ extracts identified from *in vitro* experiments. The animal experiment was designed as shown in Table 2.

**Table 2:** Grouping of animal experiments.

Group	Number	Vaccine	Vaccine plus adjuvant
A (Control Group)	6	−	−
B (Vaccine Group)	6	+	−
C (Vaccine plus Extract Group)	6	+	+

“−” indicates absence; “+” indicates presence.

Each group consisted of six randomly selected 4-week-old piglets (All piglets were purchased from Chengdu Wangjiang Agriculture and Animal Husbandry Technology Co., Ltd., and the weight variance among them in the study was within a range of 500 g). After testing negative for both PRRSV antibodies and antigens, all piglets were fed and watered normally. Piglets in groups B and C were vaccinated at 4 weeks, with the dosage and administration method following the manufacturer’s instructions. Group A received an equivalent dose of vaccine diluent. The study was conducted using a double-blind design, ensuring that neither the researchers administering the treatments nor those assessing the outcomes were aware of the group assignments.

For group C, the CZ-AM extract was added to the feed. The reference to Astragalus and Curcuma slices is based on the guidelines from the Chinese Veterinary Pharmacopoeia (China Veterinary Pharmacopoeia Committee, 2020), which state that the daily intake of Astragalus slices for each pig should not exceed 15 g, and the daily intake of Curcuma slices should not exceed 10 g. In the preparation of the extract, approximately 1 g of extract was derived from 3 g of slice. Therefore, according to the guidelines, the daily intake of the AM extract for each pig should not exceed 5 g, and the CZ extract should not exceed 3.3 g.

#### 2.4.1 Clinical symptom observation

Daily monitoring of the piglets was conducted to assess their health status and assign clinical scores based on behavior, respiratory symptoms, and coughing. The clinical symptom scoring criteria are detailed in Supplementary Table S2. Rectal temperature was observed and recorded daily. All piglets were weighed at the beginning and end of the experiment to calculate the average daily weight gain. The experiment concluded 28 days after vaccination (Table 2).

#### 2.4.2 PRRSV antibody and cytokine ELISA detection

Blood samples were collected from the anterior vena cava of piglets at 0, 7-, 14-, 21-, and 28-days post-vaccination. The PRRSV N protein-specific antibodies were detected using an ELISA kit (purchased from Shenzhen Kangbaide Biotechnology Co., Ltd., Shenzhen, China). The secretion levels of cytokines IL-1*β*, IL-6, IL-4, TNF-*α*, and IL-2 were measured using ELISA kits (purchased from Chongqing Bonuoheng Biotechnology Co., Ltd., Chongqing, China), following the instructions provided with the kits.

#### 2.4.3 Regulation of NLRP3 inflammasome mRNA by the CZ-AM extract in porcine lungs

Total RNA was extracted from porcine lung tissue, and reverse transcribed into cDNA. mRNA levels of TLR4, MyD88, NLRP3, ASC, and Caspase-1 were quantified by RT-qPCR, with β-actin as the internal control. Relative expression levels were calculated using the 2^−ΔΔCT^ method. Primers were designed using Primer 5, with details provided in Supplementary Table S3.

#### 2.4.4 Porcine peripheral blood lymphocyte proliferation assay

Blood samples were collected from piglets at 7-, 14-, 21-, and 28-days post-vaccination. The proliferation of porcine peripheral blood lymphocytes was assessed using the method described by Shao (2009). Lipopolysaccharide (LPS) and concanavalin A (ConA) (purchased from Sigma-Aldrich Trading Co. Ltd., USA) were used as antigenic stimulants. After stimulation, MTT reagent was added to each well, and metabolically active lymphocytes reduced the MTT to formazan crystals. The crystals were dissolved, and the optical density (OD) at 570 nm for each well was measured to evaluate the lymphocyte proliferation.

### 2.5 Data analysis

All statistical analyses were performed using SPSS 18.0 with one-way analysis of variance (ANOVA). A *p*-value of <0.05 was considered the threshold for statistical significance.

## 3 Results

### 3.1 Determination of TCID50 for PRRSV SCCD22

The PRRSV viral fluid was diluted tenfold and inoculated onto the 96-well cell culture plate. After incubating for 5 days at 37°C in a CO_2_ atmosphere, the TCID_50_ of PRRSV was calculated using the Reed-Muench method. As indicated in Supplementary Table S4, the TCID_50_ for the PRRSV SCCD22 was determined to be 10^–6.39^/0.1 mL.

### 3.2 Effects of different concentrations of extracts on Pams-163

**Figure 1:**
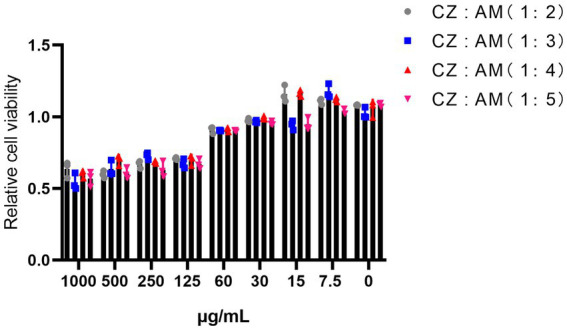
Relative viability of Pams-163 under different ratios and concentrations of the CZ-AM extracts.

The results showed that when the two extracts were combined (Figure 1), Pams-163 viability was ≥0.9 at extract concentrations ≤60 μg/mL. Therefore, the maximum safe concentration for all ratios of CZ-AM extracts was determined to be 60 μg/mL.

**Figure 2:**
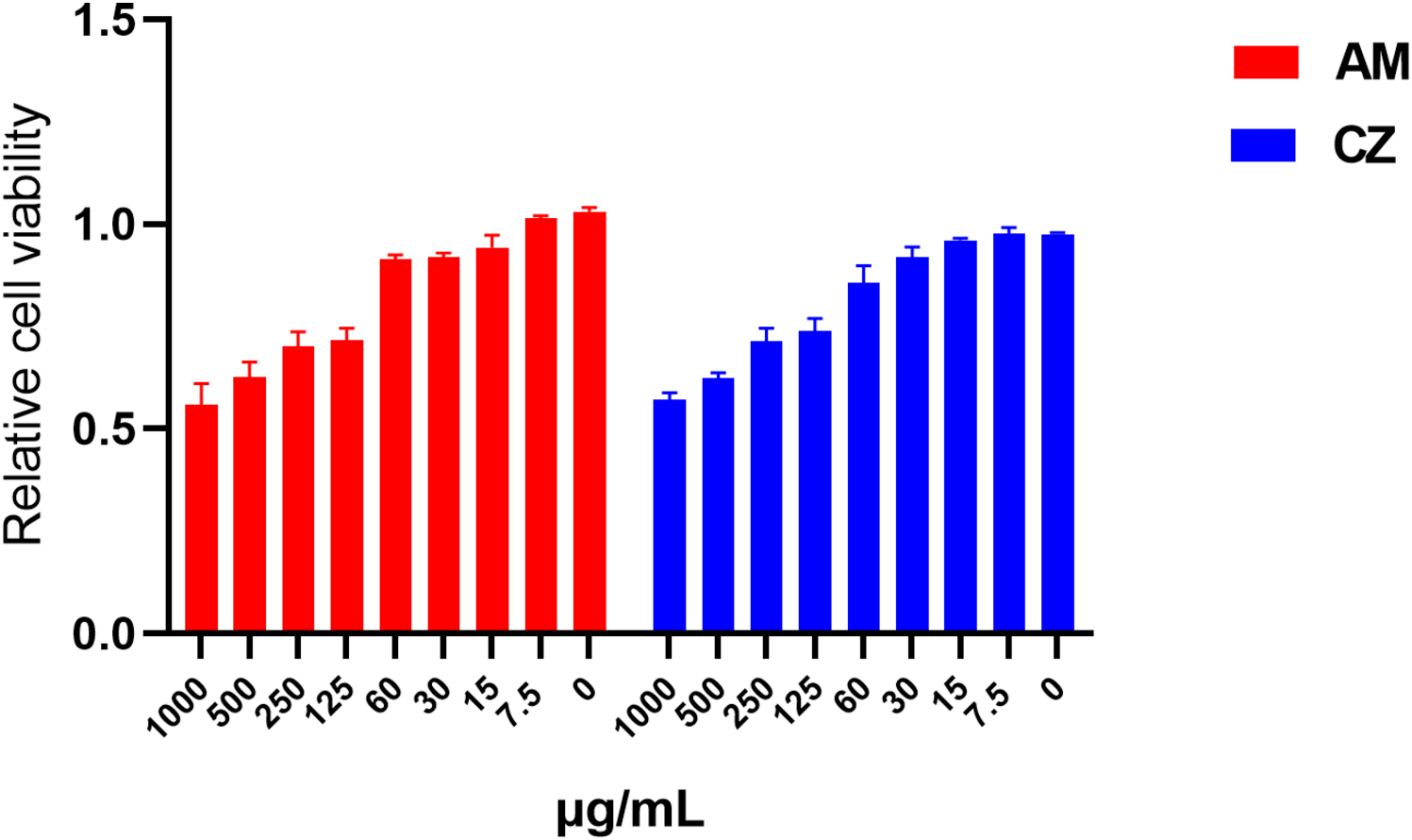
Relative viability of Pams-163 under individual treatment with the AM extract and CZ extract.

When the extracts were used individually (Figure 2). For the AM extract, Pams-163 viability was ≥0.9 at concentrations ≤60 μg/mL, indicating that the maximum safe concentration for the AM extract was 60 μg/mL. For the CZ extract, Pams-163 viability was ≥0.9 at concentrations ≤30 μg/mL, indicating that the maximum safe concentration for the CZ extract was 30 μg/mL.

### 3.3 Optimal mode of action of extracts against PRRSV

#### 3.3.1 Mode of action of the CZ-AM extract against PRRSV

**Figure 3:**
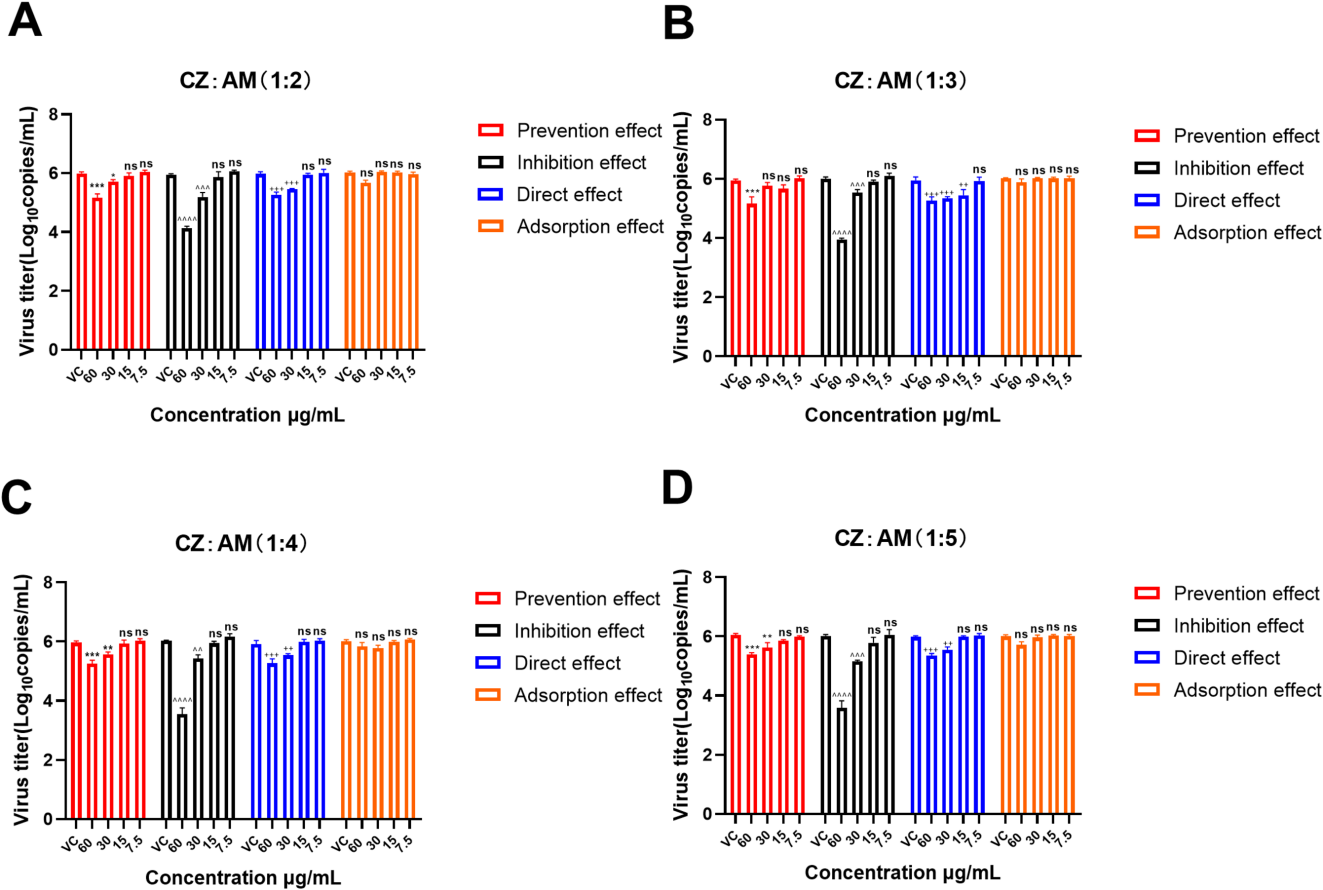
Mode of action of different ratios of the CZ-AM extract against PRRSV. **(A–D)** correspond to the different ratios of CZ: AM (1:2, 1:3, 1:4, 1:5). Viral copies are presented as mean ± standard deviation (each sample in triplicate). VC, Virus control group. Significance was analyzed using Pearson *r*^2^. Comparisons to the virus control group are indicated as follows: Not significant: ns (*p* > 0.05); Significant: */^/+ (*p* < 0.05); Very significant: **/^^/++ (*p* < 0.01); Highly significant: ***/^^^/+++ (*p* < 0.001); Extremely significant: ****/^^^^/++++ (*p* < 0.0001).

In this study, concentration gradients of 60 μg/mL, 30 μg/mL, 15 μg/mL, and 7.5 μg/mL were used to screen the mode of action of the CZ-AM extract. The results (Figure 3) indicated that adsorption did not significantly inhibit PRRSV in all ratios (*p* > 0.05). Among the other three modes of action, all extract ratios at a concentration of 60 μg/mL maximally reduced PRRSV copies.

Specifically, the inhibition effect with 60 μg/mL of the CZ-AM extract resulted in the lowest viral copy numbers (0.21 ~ 1.37 × 10^4^ copies/mL) and the most significant reduction (*p* < 0.0001). This outcome was superior to the viral copies observed under the prevention effect (11.17 ~ 31.99 × 10^4^ copies/mL) and direct effect (12.93 ~ 26.32 × 10^4^ copies/mL) at the same concentration. This indicates that the CZ-AM extract has a significant antiviral effect *in vitro*, with the inhibition effect being the optimal mode of action.

Additionally, within the inhibition effect, when the concentration was 60 μg/mL and the ratio of CZ to AM was 1:4 or 1:5, the viral load was significantly lower than other ratios, with the lowest sample viral load being approximately 0.21 × 10^4^ copies/mL.

#### 3.3.2 Optimal mode of action of individual AM extract and CZ extract against PRRSV

**Figure 4:**
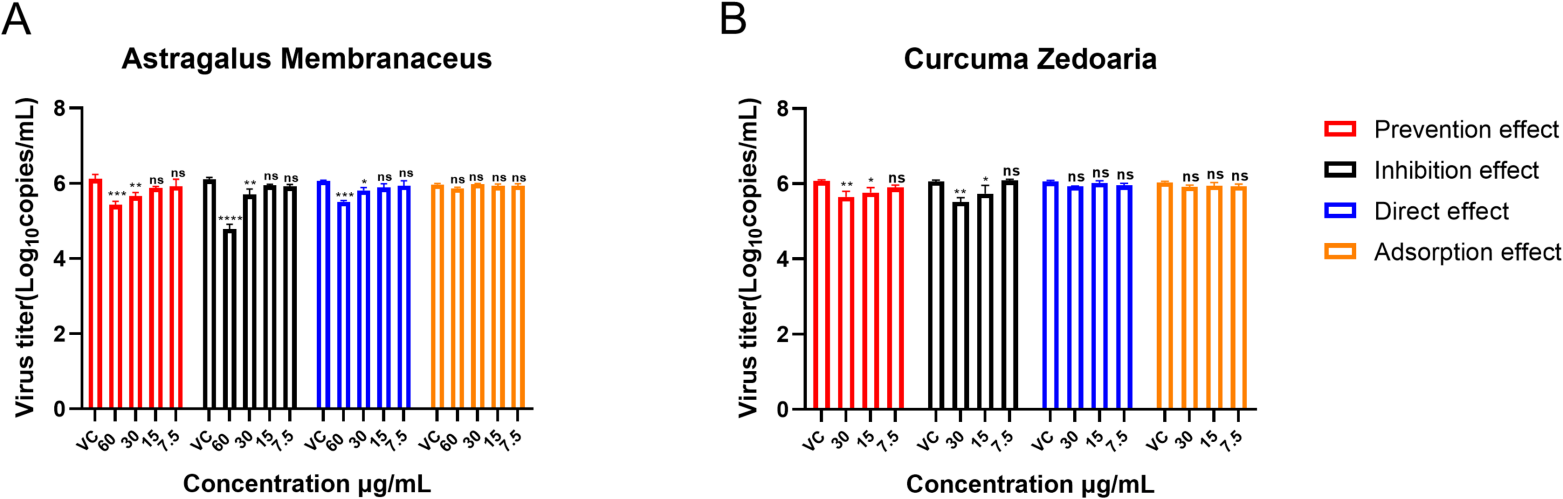
Mode of action of the AM extract and CZ extract against PRRSV. **(A and B)** are the results of the AM extract and CZ extract against PRRSV, respectively. Viral copies are presented as mean ± standard deviation (each sample in triplicate). VC, Virus Control Group. Significance was analyzed using Pearson *r*^2^. Comparisons to the virus control group are indicated as follows: Not significant: ns (*p* > 0.05); Significant: * (*p* < 0.05); Very significant: ** (*p* < 0.01); Highly significant: *** (*p* < 0.001); Extremely significant: **** (*p* < 0.0001).

The AM extract was screened at concentrations of 60 μg/mL, 30 μg/mL, 15 μg/mL, and 7.5 μg/mL. The CZ extract was screened at concentrations of 30 μg/mL, 15 μg/mL, and 7.5 μg/mL. The results (Figure 4) showed that the adsorption effect of both AM and CZ extracts on Pams-163 did not significantly inhibit PRRSV (*p* > 0.05). When used in the inhibition effect, the AM extract at 60 μg/mL and Curcuma extract at 30 μg/mL resulted in the greatest reduction of PRRSV viral copies, with the lowest viral copiers being 4.27 ~ 7.41 × 10^4^ copies/mL for the AM extract and 2.57 ~ 4.27 × 10^5^ copies/mL for the CZ extract. These results indicate that both AM and CZ extracts have significant antiviral effects *in vitro*, with the inhibition effect being the most effective.

### 3.4 Detection relative mRNA expression of cytokines

#### 3.4.1 Detection relative mRNA expression of cytokines in Pams-163 after treatment with the CZ-AM extracts

**Figure 5:**
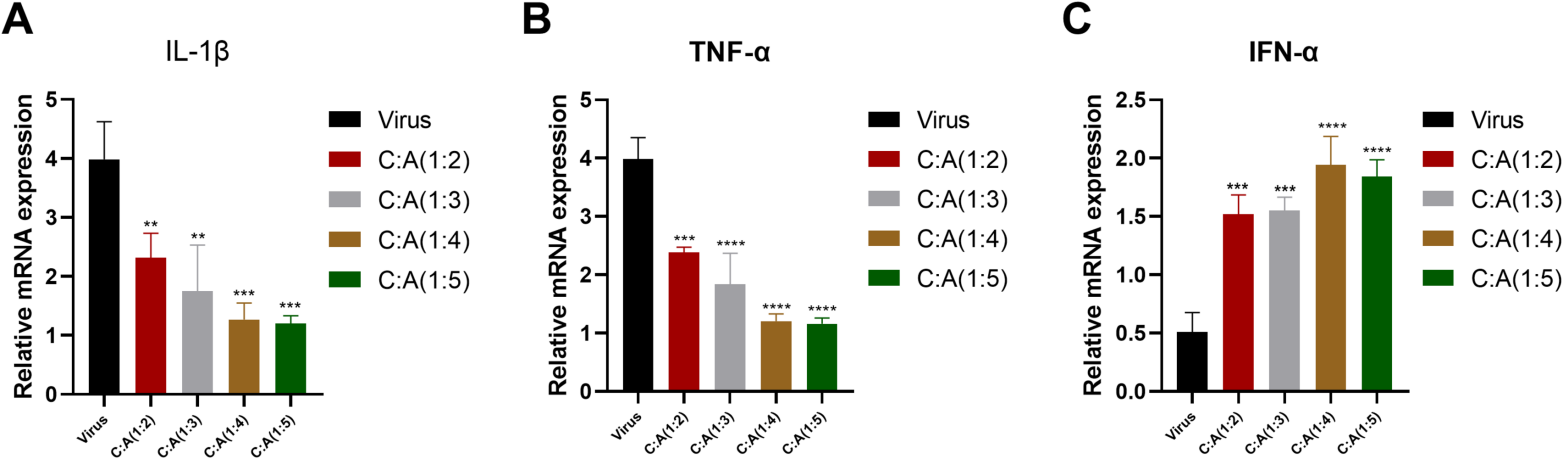
Relative mRNA expression of cytokine mRNA in Pams-163 after extract treatment and PRRSV inhibition. **(A–C)** are the results of IL-1β, TNF-α, and IFN-α, respectively. Significance was analyzed using Pearson *r*^2^. Comparisons to the virus control group are indicated as follows: Not significant: ns (*p* > 0.05); Significant: * (*p* < 0.05); Very significant: ** (*p* < 0.01); Highly significant: *** (*p* < 0.001); Extremely significant: **** (*p* < 0.0001).

This study used the inhibition effect of the CZ-AM extracts (60 μg/mL) against PRRSV. After 24 h of treatment, the Ct values of IL-1*β*, TNF-*α*, IFN-α, and porcine β-actin in Pams-163 were measured. The results were calculated using the 2^−ΔΔCt^ method. The results (Figure 5) showed that, compared to the virus control group: The relative mRNA expression of TNF-α significantly decreased in all ratios of CZ-AM extracts extracts, with the most significant reduction observed at ratios of 1:4 and 1:5; The relative mRNA expression of IL-1β significantly decreased in all ratios of CZ-AM extracts (*p* < 0.01), with the most significant reduction observed at ratios of 1:4 and 1:5; When the ratio of CZ to AM in the extracts is between 1:2 and 1:5, the relative mRNA expression of IFN-*α* can be significantly upregulated, with the most notable increase observed at a ratio of 1:4.

In conclusion, when the ratio of CZ to AM in the extract is 1:4 or 1:5, the pro-inflammatory cytokines IL-1β and TNF-α are maximally suppressed. Additionally, the ratio of 1:4 results in the highest levels of IFN-α. Therefore, the optimal ratio of CZ to AM in the extracts, as determined by the *in vitro* experiments, is 1:4.

#### 3.4.2 Detection relative mRNA expression of cytokines in Pams-163 after treatment with individual AM extract and CZ extract

**Figure 6:**
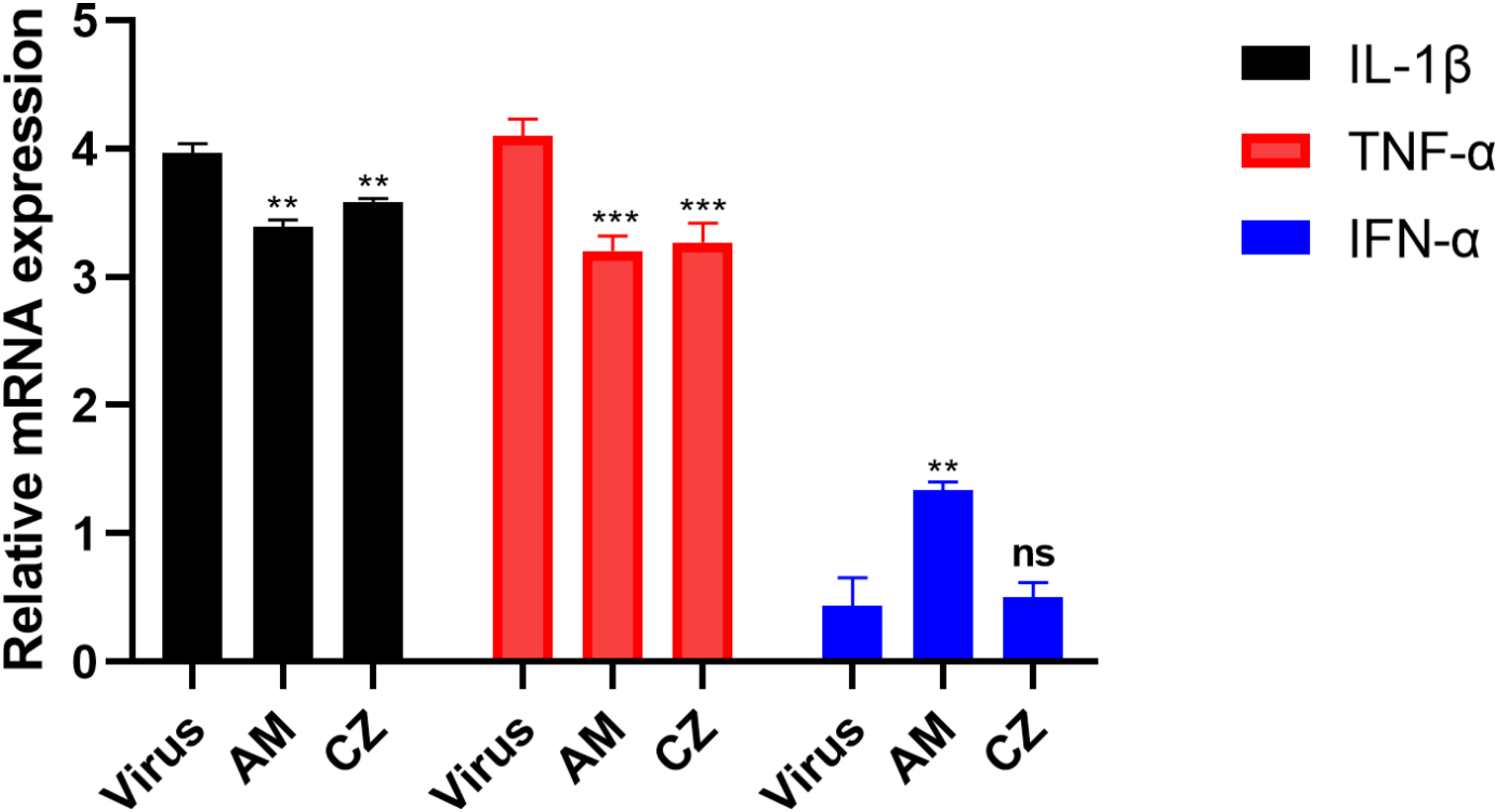
Relative mRNA expression of cytokine mRNA After inhibition of PRRSV by individual extracts. Significance was analyzed using Pearson *r*^2^. Comparisons to the virus control group are indicated as follows: Not significant: ns (*p* > 0.05); Significant: * (*p* < 0.05); Very significant: ** (*p* < 0.01); Highly significant: *** (*p* < 0.001); Extremely significant: **** (*p* < 0.0001).

The results (Figure 6) showed that, compared to the virus group, both AM and CZ extracts significantly reduced the relative mRNA expression of IL-1β and TNF-*α*. Additionally, the AM extract significantly upregulated the relative mRNA expression of IFN-*α*, whereas the CZ extract showed no significant difference compared to the virus group.

In conclusion, by comparing the antiviral and immunomodulatory effects of the combined and individual actions of the AM extract and CZ extract *in vitro*, this study found the following: Under the same MOI of PRRSV infection, both combined and individual actions of two extracts significantly inhibited PRRSV replication *in vitro*. However, the combined action resulted in the lowest PRRSV copies (0.21 ~ 1.37 × 104 copies/mL), while the individual actions of the extracts resulted in minimum PRRSV copies of 4.27 ~ 7.41 × 104 copies/mL copies/mL (AM extract) and 2.57 ~ 4.27 × 105 copies/mL (CZ extract). This indicates that the combined extracts significantly enhance the antiviral effect, surpassing the efficacy of either extract used alone; Both the combined and individual actions of the extracts significantly downregulated IL-1β and TNF-*α* mRNA levels *in vitro*. However, the combined action of AM and CZ extracts significantly upregulated IFN-α mRNA levels, whereas the individual action of the CZ extract did not result in significant changes in IFN-α mRNA levels.

### 3.5 Determination results of Astragaloside IV and Curcuma volatile oil in the CZ-AM extract

#### 3.5.1 Determination results of Astragaloside IV

The concentration of Astragaloside IV in the CZ-AM extract was determined using HPLC. The results are as follows:

The standard solution of Astragaloside IV was injected into the HPLC at volumes of 8 μL, 10 μL, 12 μL, 14 μL, and 16 μL. The chromatogram of the standard solution is shown in Supplementary Figure S1, and the results are presented in Table 3. The standard curve was obtained with the equation: Y (peak area) = 70.434X (injection volume) − 217.22.

**Table 3:** Determination results of Astragaloside IV standard solution.

The volume of the sample (μL)	Astragaloside iv (μg)	Standard concentration (mg/mL)	Peak area
8	8	1.0	347.1492
10	10		490.9958
12	12		621.9656
14	14		765.6982
16	16		914.1419

**Figure 7:**
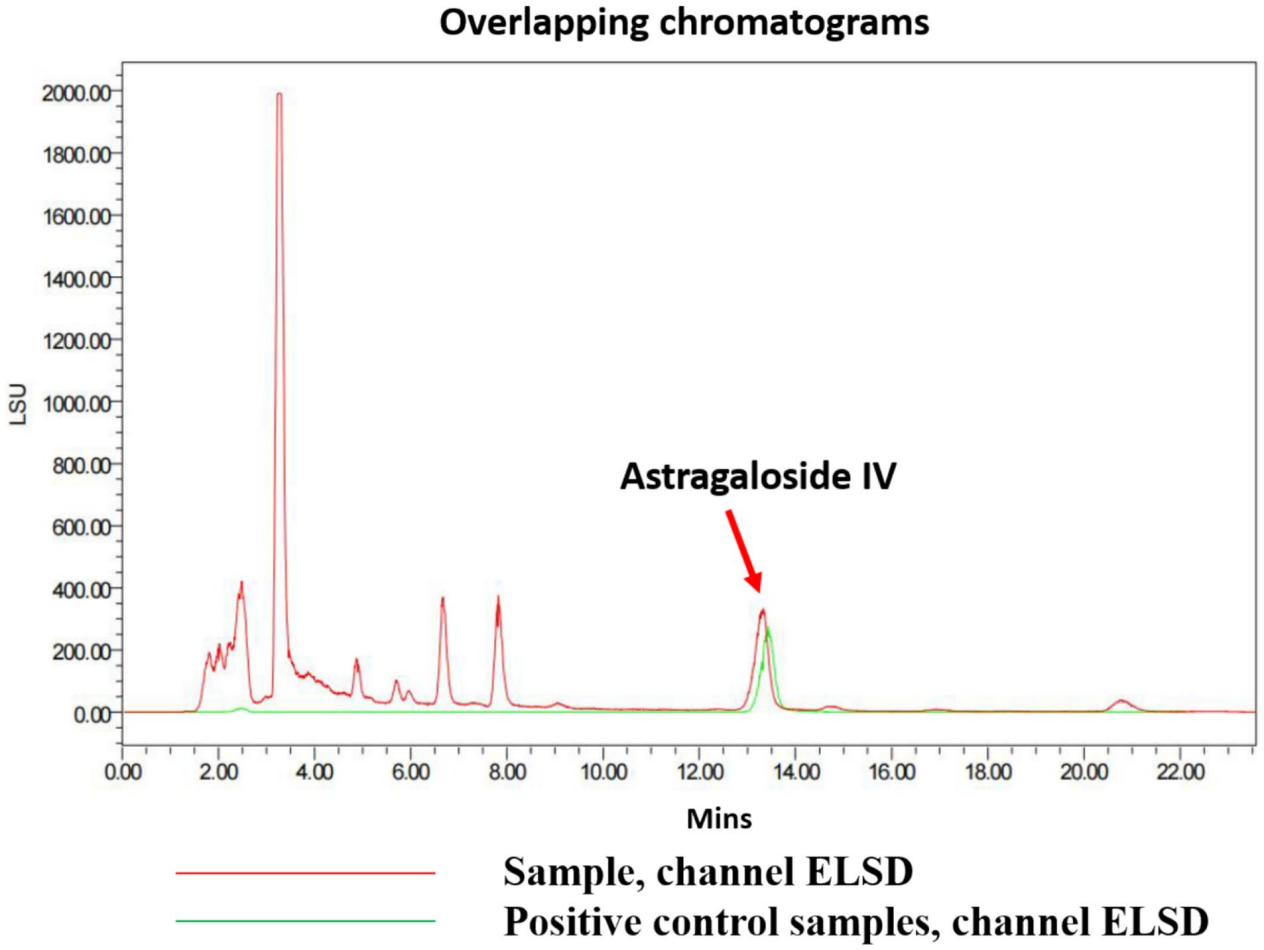
Chromatogram of Astragaloside IV in the test sample.

The determination results for 6 g of the test sample containing Astragaloside IV are shown in Figure 7. Under the chromatographic conditions, all components achieved baseline separation, and the test sample had one peak coinciding with the Astragaloside IV standard. In this study, the peak area measured for Astragaloside IV was 620.6519. Using the standard curve equation, the corresponding injection volume (X) was calculated to be 11.9 μL. Based on the sample concentration of 1,200 mg/mL, the total mass of Astragaloside IV was calculated to be 14,280 μg. Therefore, the Astragaloside IV content in the sample was 0.238% (14,280 μg/6 g). This value exceeds the requirement of the Chinese Pharmacopoeia (2020 edition), which specifies that the Astragaloside IV content (C41H68O14) should not be less than 0.080%.

#### 3.5.2 Determination results of Curcuma volatile oil

The content of Curcuma volatile oil in the extracts was determined using the distillation method with a distillation apparatus set up in our laboratory. The volatile oil content was found to be 1.6%, which is higher than the minimum requirement of 1.0% specified in the Chinese Pharmacopoeia (2020 edition).

### 3.6 Preliminary investigation of the CZ-AM extract as PRRSV vaccine adjuvants for piglets

The above studies have shown that the CZ-AM extract exhibit both antiviral activity and immunomodulatory effects *in vitro*, outperforming the effects of each extract used individually. Based on these findings, this study further explored the potential of the CZ-AM extract as adjuvants for PRRSV inactivated vaccines in piglets.

#### 3.6.1 Clinical symptom observation results

**Figure 8:**
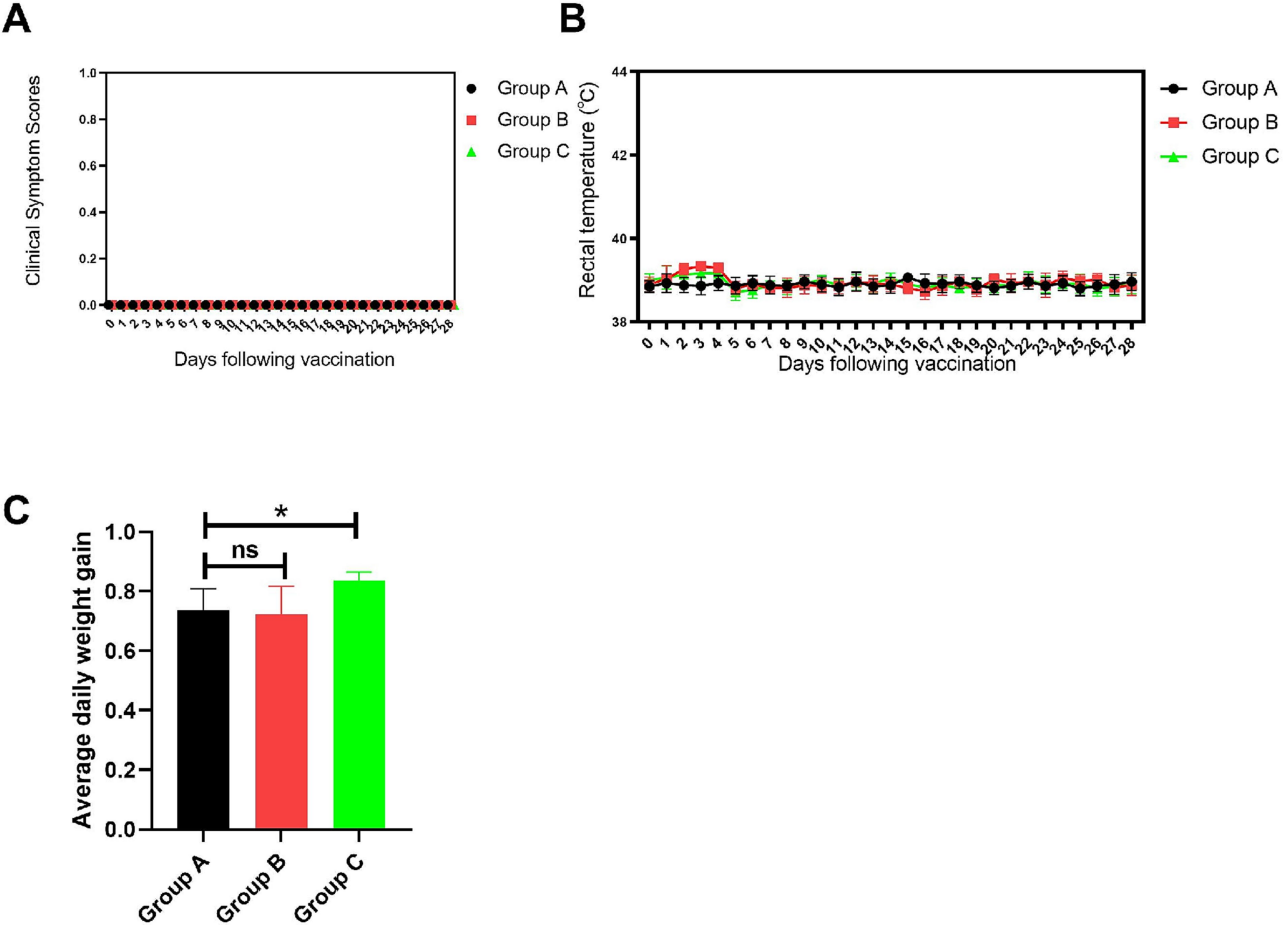
Clinical symptom observations. **(A)** Clinical symptom scores. **(B)** Rectal temperature statistics. **(C)** Average daily weight gain statistics. Comparisons to the group A are indicated as follows: Not significant: ns (*p* > 0.05); Significant: * (*p* < 0.05).

The clinical symptom scores (Figure 8A) indicated that after vaccination, piglets in the blank control group (Group A), the vaccine group (Group B), and the vaccine plus extract group (Group C) did not show any clinical symptoms. However, the body temperature of piglets in Groups B and C slightly increased after vaccination (Figure 8B), peaking on the fourth day. This temperature rise might be an immune response to the vaccination. Over time, the body temperatures of piglets in Groups B and C returned to similar levels, with no significant differences between the groups. This suggests that, in the long term, vaccination and extract supplementation do not affect the body temperature and clinical symptoms of piglets. And the results of the average daily weight gain (Figure 8C) showed that, compared to the control group, the piglets in Group C had a significantly higher average daily weight gain (*p* < 0.05).

#### 3.6.2 Results of PRRSV antibody levels and cytokine secretion levels

**Figure 9:**
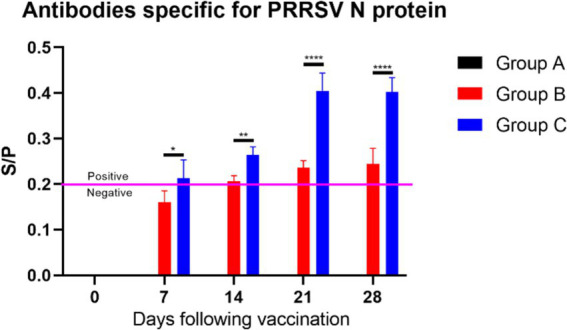
S/P ratio statistics of PRRSV N Protein antibody levels. When the sample S/P ratio is ≥0.2, it is considered PRRSV antibody positive. Comparisons of significant differences between group B and group C are represented as follows: Significant: * (*p* < 0.05); Very significant: ** (*p* < 0.01); Extremely significant: **** (*p* < 0.0001).

The PRRSV antibody results (Figure 9) showed that: In the blank control group (Group A), no PRRSV antibodies were detected, indicating no interference from PRRSV during the experiment and confirming that the study started with healthy, virus-free piglets; In the vaccine group (Group B), PRRSV antibody levels gradually increased over time. By day 14 post-vaccination, the average S/P ratio of PRRSV antibodies in the piglets’ serum was greater than 0.2, and by day 21, all piglets in Group B tested positive for PRRSV antibodies; In the vaccine plus extract group (Group C), PRRSV antibody levels were significantly higher than those in Group B starting from day 7 post-vaccination (*p* ≤ 0.05). The antibody levels in Group C peaked on day 21, with the difference in antibody levels between Groups B and C being greatest on days 21 and 28 (*p* ≤ 0.0001). These findings suggest that the CZ-AM extract may enhance the immune response of piglets to PRRSV, indicating their potential as a beneficial immune-boosting strategy.

**Figure 10:**
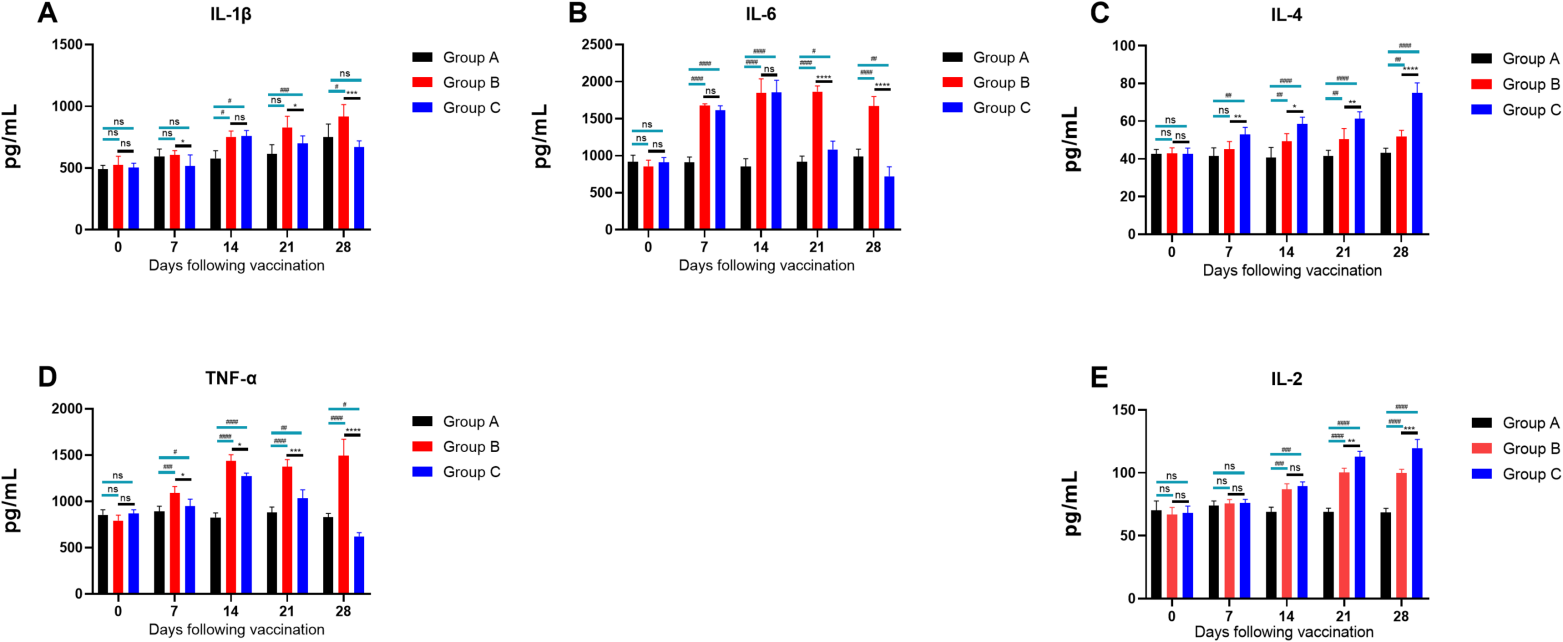
Cytokine secretion levels in piglets. **(A–E)** are the results of IL-1β, IL-6, IL-4, TNF-α, and IL-2, respectively Significance was analyzed using Pearson *r*^2^. The levels of significance are indicated as follows: Not significant: ns (*p* > 0.05); Significant: */# (*p* < 0.05); Very significant: **/## (*p* < 0.01); Highly significant: ***/### (*p* < 0.001); Extremely significant: ****/#### (*p* < 0.0001).

The results of cytokine secretion levels in piglets (Figure 10) indicated the following: The levels of IL-1β (Figure 10A) in Groups B and C generally showed an upward trend within 14 days. By day 21, IL-1β levels in Group C were significantly lower than in Group B (*p* ≤ 0.05), indicating that feeding the CZ-AM extract can significantly reduce IL-1β secretion 14 days post-vaccination; The levels of IL-6 (Figure 10B) in Groups B and C generally showed an upward trend within 14 days. From day 21, IL-6 levels in Group C were significantly lower than in Group B (*p* ≤ 0.0001), indicating that feeding the CZ-AM extracts can significantly reduce IL-6 secretion starting from 14 days post-vaccination; Starting from day 7 post-vaccination, IL-4 levels (Figure 10C) in Group C significantly increased over time compared to Group B, indicating that feeding the CZ-AM extract can significantly enhance IL-4 secretion post-vaccination; The levels of TNF-*α* (Figure 10D) in Groups B and C showed an upward trend within 14 days. From day 7, TNF-α levels in Group C were significantly lower than in Group B, reaching the lowest point on day 28. This indicates that feeding the CZ-AM extract can significantly reduce TNF-α secretion starting from 7 days post-vaccination; IL-2 levels (Figure 10E) in Groups B and C significantly increased from day 14 (*p* ≤ 0.001). By day 21, IL-2 levels in Group C were significantly higher than in Group B, indicating that feeding the CZ-AM extract can significantly increase IL-2 secretion starting from 14 days post-vaccination.

#### 3.6.3 Regulation of NLRP3 inflammasome mRNA by the CZ-AM extract in porcine lungs

**Figure 11:**
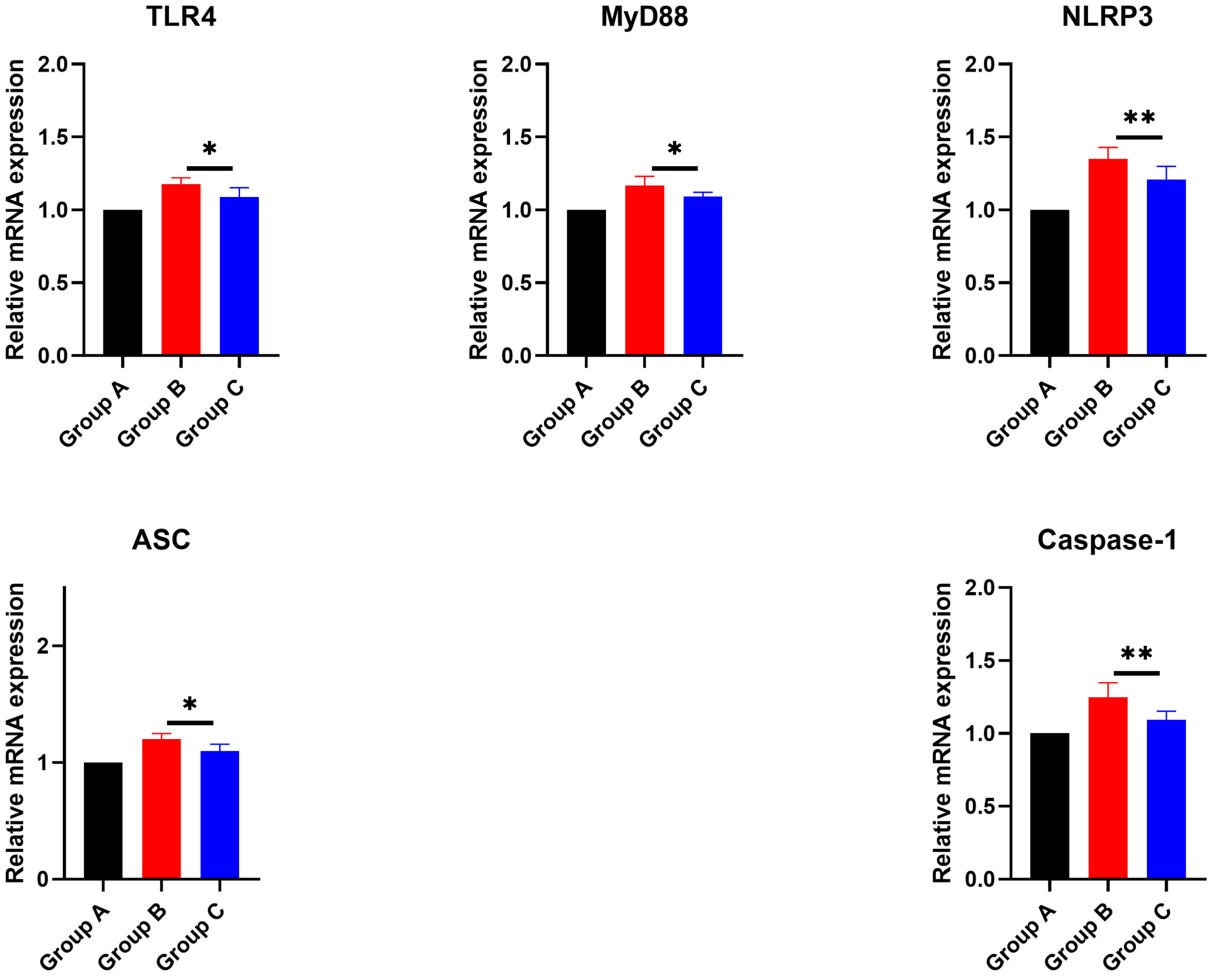
Effects of the CZ-AM extract on the mRNA expression of inflammation-related genes in porcine lung tissue. * and ** indicate significant (*p* ≤ 0.05) and very significant (*p* ≤ 0.01) comparison between group C and group B, respectively.

Previous experiments demonstrated that the injection of inactivated PRRSV vaccine significantly increased the secretion of IL-1β, IL-6, and TNF-α, while feeding the CZ-AM extract notably reduced these cytokine levels. Moreover, we analyzed the mRNA expression of genes in the TLR4/NLRP3/IL-1β signaling pathway (TLR4, MyD88, NLRP3, ASC, and Caspase-1). Results showed that (Figure 11) compared to Group B (vaccine group), mRNA levels of these genes were significantly reduced in Group C (vaccine plus extract) (*p* ≤ 0.05). These findings suggest that the CZ-AM extract exerts anti-inflammatory effects, potentially through modulation of the TLR4/NLRP3/IL-1β pathway.

#### 3.6.4 Results of peripheral blood lymphocyte proliferation assay in piglets

The results of the ConA-stimulated T lymphocyte proliferation assay for the CZ-AM extract in piglets (Table 4) indicated the following: In the vaccine plus extract group (Group C), the OD values started to increase from day 7, reaching the highest point on day 21, which coincided with the peak antibody levels. The OD values remained above 0.6 thereafter; The proliferation rate of T lymphocytes in Group C was significantly higher than that in the vaccine group (Group B) from days 7 to 28 post-vaccination (0.001 < *p* ≤ 0.01).

**Table 4:** Effect of ConA stimulation on proliferation of peripheral blood lymphocytes in piglets (OD570 nm values).

Days	OD570 nm values		
	A (Blank control group)	B (Vaccine group)	C (Vaccine plus extract Group)
7	0.223 ± 0.034	0.256 ± 0.006 ^ns^	0.429 ± 0.016**^##^
14	0.31 ± 0.024	0.394 ± 0.024*	0.583 ± 0.035**^##^
21	0.252 ± 0.026	0.509 ± 0.011**	0.656 ± 0.016***^##^
28	0.243 ± 0.018	0.487 ± 0.037**	0.632 ± 0.017***^##^

ns, *, **, *** indicate no significant difference compared to Group A (*p* > 0.05), significant difference (*p* ≤ 0.05), very significant difference (0.001 < *p* ≤ 0.01), and extremely significant difference (*p* ≤ 0.001), respectively. ## indicates a very significant difference compared to Group B (0.001 < *p* ≤ 0.01).

The results of the LPS-stimulated B lymphocyte proliferation assay for the CZ-AM extract in piglets (Table 5) indicated the following:In the vaccine plus extract group (Group C), the OD values reached their highest point on day 21, coinciding with the peak antibody levels, and then slightly decreased; The proliferation rate of B lymphocytes in Group C was significantly higher than that in the vaccine group (Group B) from days 7 to 28 post-vaccination (0.001 < *p* ≤ 0.05).

**Table 5:** Effect of LPS stimulation on proliferation of peripheral blood lymphocytes in piglets (OD570 nm values).

Days	OD570 nm values		
	A (Blank control group)	B (Vaccine group)	C (Vaccine plus extract Group)
7	0.227 ± 0.036	0.228 ± 0.041^ns^	0.461 ± 0.033**^##^
14	0.255 ± 0.018	0.394 ± 0.011*	0.665 ± 0.022***^##^
21	0.314 ± 0.013	0.482 ± 0.035*	0.709 ± 0.006***^##^
28	0.291 ± 0.012	0.524 ± 0.023**	0.65 ± 0.026***^#^

ns, *, **, *** indicate no significant difference compared to Group A (*p* > 0.05), significant difference (*p* ≤ 0.05), very significant difference (0.001 < *p* ≤ 0.01), and extremely significant difference (*p* ≤ 0.001), respectively. #, ## indicate significant difference compared to Group B (0.01 < *p* ≤ 0.05) and very significant difference (0.001 < *p* ≤ 0.01), respectively.

## 4 Discussion

In our study, we first screened the optimal mode of action and concentration of the CZ-AM extract on Pams-163 against PRRSV. The results indicated that the extract exhibited an inhibitory effect on PRRSV *in vitro*. Specifically, when the ratio of CZ to AM in the extract was 1:4 or 1:5, it significantly inhibited PRRSV and the pro-inflammatory cytokines IL-1β and TNF-*α*. Additionally, the 1:4 ratio most notably upregulated the relative mRNA expression of IFN-*α*.

Next, we investigated the differences between the effects of the AM extract and CZ extract when extracted separately and together. The results showed that, under the same MOI PRRSV inoculation, the CZ-AM extract significantly inhibited PRRSV replication *in vitro*, outperforming either extract used alone. In terms of immunomodulation, both extracts, whether used individually or in combination, significantly downregulated the relative mRNA expression of IL-1β and TNF-*α*, indicating their anti-inflammatory properties. Notably, the CZ-AM extract significantly upregulated IFN-α mRNA expression, whereas Curcuma extract alone had no significant effect on IFN-*α*. This finding suggests that the combined extract of AM and CZ not only has a stronger antiviral capability but also enhances the host’s antiviral effect and immune response by modulating the expression of immune factors. The enhanced antiviral and immune effects may be related to the combined decoction of AM and CZ, which promotes the dissolution of their active components (Yin et al., 2018; Wu et al., 2018), such as astragaloside, astragalosaponin I and II, calycosin, flavonoids, curcumenol, curdione, isocurcumenol, furanodiene, zedoarondiol, germacrone, etc.

IL-1β is a potent pro-inflammatory cytokine primarily secreted by lymphocytes, macrophages, and monocytes. During viral infections or inflammation, the increased expression of pattern recognition receptors (PRRs) and Toll-like receptors (TLRs) leads to enhanced IL-1β expression. IL-1β stimulates CD4+ cells to differentiate into Th17 cells (Turner et al., 2014). Similar to other Th1 pro-inflammatory cytokines, TNF-*α* plays a crucial role in both local and systemic inflammatory responses (Zlotnik and Yoshie, 2012; Tay et al., 2020). The downregulation of IL-1β and TNF-α in PRRSV-infected Pams-163 cells by the CZ-AM extract indicates its anti-inflammatory properties. In addition, this study demonstrated that the CZ-AM extract inhibited PRRSV replication and upregulated IFN-α expression in Pams-163. IFN-α primarily participates in antiviral processes by increasing the expression of IL-2 receptors on helper T lymphocytes, thereby enhancing the production and function of CD8 cytotoxic T cells (Bramson et al., 2003). IFN-α is involved in both innate and adaptive immune responses, enhancing the antibody response to soluble antigens (Santini et al., 2000). It also increases the expression of type I and type II MHC molecules, induces the production of IgE and IgG antibodies, and enhances antigen presentation, thereby boosting the immune response (Kim et al., 2001). Previous studies have reported that PRRSV can inhibit the synthesis of type I IFNs in pigs. IFN-α is almost undetectable in the lungs of pigs with extensive PRRSV replication, and porcine alveolar macrophages infected with PRRSV do not produce IFN-α (Albina et al., 1998). In this study, the CZ-AM extract not only inhibited PRRSV replication in Pams-163 cells but also upregulated IFN-*α* expression. This indicates that the extract can activate porcine alveolar macrophages, promoting the production of IFN-α, which helps to inhibit viral proliferation.

In addition, the content of Astragaloside IV and curcuma volatile oil in the extract prepared in this study met the requirements for active ingredient content as stipulated in the Chinese Pharmacopoeia (2020) (National Pharmacopoeia Commission, 2020). However, the curcuma volatile oil content in this study was lower than the yield rates of 2.5–5% reported in other literature (Lei et al., 2003; Li et al., 2005; Wang et al., 2008). The lower content of curcuma volatile oil may be due to the traditional distillation method we used, which requires continuous heating. Given the volatile nature of curcuma oil, a portion may evaporate with the steam over the prolonged distillation process. Nonetheless, the distillation method has the advantages of being simple to operate and highly reproducible, requiring only conventional distillation equipment and basic operational skills.

The findings showed that the CZ-AM extract exhibited positive effects *in vitro*, demonstrating both antiviral activity and the ability to modulate immune responses. To address the common issue of poor immune efficacy of PRRSV inactivated vaccines, we further investigated the potential of the CZ-AM extract as an adjuvant for the PRRSV inactivated vaccine (CH-1a strain) in piglets.

The results indicated that feeding the CZ-AM extract had no impact on the body temperature and clinical symptoms of piglets, demonstrating good safety. Additionally, it significantly increased the body weight of the piglets. The PRRSV inactivated vaccine (CH-1a strain) in piglets led to an increase in the secretion of IL-1β, IL-6, and TNF-*α*. However, over time, the CZ-AM extract significantly reduced the secretion of IL-1β, IL-6, TNF-α and increased the secretion of IL-4 and IL-2, thereby mitigating the inflammatory response. Furthermore, using the CZ-AM extract as a vaccine adjuvant resulted in the highest antibody levels 21 days post-vaccination, which were significantly higher than those in the group that received the vaccine alone.

As previously mentioned, IL-1β and TNF-*α* are potent pro-inflammatory cytokines. Studies have shown that pro-inflammatory cytokines TNF-α and IL-1 are key mediators of the inflammatory response caused by various infectious pulmonary diseases, and increased levels of these cytokines lead to systemic acute inflammation (Murtaugh et al., 1996). IL-4 promotes B cell proliferation and differentiation and enhances the functions of macrophages and Tc cells. IL-6 promotes B cell differentiation and antibody production, assists in Tc cell maturation, and induces the production of acute-phase proteins (APP) by hepatocytes (Shi et al., 2010). IL-2, a Th1 cytokine, stimulates the growth and proliferation of T and B cells, activates T cells to secrete IFN, enhances the cytotoxic activity of natural killer cells and macrophages, and induces the anti-tumor and anti-infection activity of tumor-infiltrating lymphocytes, primarily exerting cell-mediated immune functions (Zeng et al., 2010). Reports (Ait-Ali et al., 2007; Du et al., 2017; Czyżewska-Dors et al., 2019) indicated that typical features of PRRSV infection in pigs include persistent viremia, increased levels of inflammatory cytokines such as IL-1β, IL-6, and TNF-*α*, NK cell dysfunction, and delayed appearance of neutralizing antibodies.

In this study, continuous feeding of the CZ-AM extract effectively reduced the secretion of IL-1β, IL-6, and TNF-α in piglets, consistent with the findings of Zhou et al. (2010) and Qiu et al. (2014), and they found that Curcuma extract could inhibit inflammatory edema and changes in capillary permeability, reducing the secretion of TNF-α, IL-1β, and IL-6 in serum and alleviating inflammation. Similarly, analysis of mRNA expression in piglet lung tissue revealed that, compared to the vaccine-only Group B, TLR4, MyD88, NLRP3, ASC, and Caspase-1 mRNA levels were significantly reduced in Group C (*p* ≤ 0.05). Combined with the decreased levels of pro-inflammatory cytokines in the ELISA results, this suggests that CZ-AM extract may exert its anti-inflammatory effects by inhibiting the activation of the TLR4/NLRP3/IL-1β signaling pathway, thereby reducing the secretion of downstream inflammatory factors. Additionally, the upregulation of IL-2 secretion by the CZ-AM extract observed in this study aligns with the findings of Jiang (2015). Jiang’s research indicated that continuous feeding of Astragalus polysaccharides after PRRSV vaccination effectively promoted IL-2 secretion in piglets. However, unlike our study, Jiang’s research found that continuous feeding of Astragalus polysaccharides did not increase antibody secretion. In this study, the PRRSV inactivated vaccine (CH-1a strain) induced an increase in the secretion of IL-1β, IL-6, and TNF-α in piglets. Over time, the CZ-AM extract significantly reduced the secretion of IL-1β, IL-6, TNF-αand increased the secretion of IL-4 and IL-2, thereby mitigating the inflammatory response. As inflammation subsided, the PRRSV antibody secretion in piglets significantly increased, indicating that the CZ-AM extract is a beneficial immunoenhancing strategy.

Finally, the lymphocyte proliferation assay in this study demonstrated that the CZ-AM extract significantly stimulated the proliferation of porcine peripheral blood T and B lymphocytes. The proliferation rate reached its peak on the 21st day after vaccination, coinciding with the highest antibody levels. This finding is consistent with Shao Shan’s (Shao, 2009) study on Astragalus as a vaccine adjuvant, which found that 24 days after injecting Astragalus polysaccharides and PRRSV inactivated vaccine, the proliferation of porcine peripheral blood lymphocytes was at its highest, as were antibody levels. This further supports the role of Astragalus components in enhancing the immune response in piglets. Additionally, Zhao’s research (Zhao, 2007) found that Astragalus polysaccharides could increase the transformation rate of porcine spleen T and B lymphocytes *in vitro*, promoting both cellular and humoral immune functions in pigs. Therefore, it can be inferred that the combined decoction of AM and CZ enhances the release of their active components, thereby promoting the proliferation and function of T and B lymphocytes, which in turn boosts the immune response in pigs.

In summary, the CZ-AM extract exhibited positive antiviral, anti-inflammatory, and immunomodulatory effects both *in vitro* and *in vivo*, highlighting its potential in the prevention and control of PRRSV. This provides a scientific basis for the development of immunoenhancing formulations based on Astragalus and Curcuma components and offers practical guidance for the modern application and development of traditional Chinese medicine formulations.

## Data Availability

The original contributions presented in the study are included in the article/Supplementary material, further inquiries can be directed to the corresponding authors.
