# Supplementary material for: In vivo and in vitro study on the combination extract of Curcuma zedoaria and Astragalus membranaceus as an adjuvant for PRRSV inactivated vaccine and its preliminary application in piglets
Source: Front Microbiol. 2024 Nov 22;15:1470297. doi: 10.3389/fmicb.2024.1470297 (PMC11622496; doi:10.3389/fmicb.2024.1470297)
Supplement: Supplementary file 1 [file Data_Sheet_1.docx]

Table S1 Information for detection primer

| Type of Virus | Sequence(5’-3’) | Target Gene | References |
| --- | --- | --- | --- |
| NADC30-like-PRRSV | F: CGTATTGGACACCTCTTTTGACTG  R: AACTGGACCTAATCTTCCTGCG | *NSP2* | (Qiu et al., 2020). |
|  | P: ROX-CCCAAAGGTCTTCGTCGGTATTCC-BHQ2 |  |  |

Table S2. Clinical symptom scoring criteria

|  | Clinical symptom | Standard for evaluation | Score | |
| --- | --- | --- | --- | --- |
|  | Body temperature | T≤39.9℃ | | 0 |
|  |  | 40.0℃≤T≤40.9℃ | | 1 |
|  |  | 41℃≤T | | 2 |
|  | Appetite | Normal | | 0 |
|  |  | Loss of appetite | | 1 |
| Gross clinical symptom score |  | Hunger strike | | 2 |
|  | State of mind | Normal | | 0 |
|  |  | Unconsciousness/coma | | 1 |
|  | Skin | Normal | | 0 |
|  |  | Cyanosis | | 1 |
| Respiratory symptom score | Respiratory symptoms | Breathes fast when nervous | | 2 |
|  |  | Shortness of breath at rest | | 3 |
|  |  | Shortness of breath and difficulty breathing at rest | | 4 |
|  |  | Severe shortness of breath, difficulty breathing | | 5 |
|  | Cough | Normal | | 0 |
|  |  | Cough | | 1 |
|  | Runny nose | Normal | | 0 |
|  |  | Runny nose | | 1 |
|  |  | Normal | | 0 |
|  |  | Shiver | | 1 |
| Neurological symptom score | Neurological symptoms | Ataxia | | 2 |
|  |  | Limbs stroke | | 3 |
|  |  | Paralysis | | 4 |

Table S3. Primers for NLRP3 inflammasoma-related genes

| Primer name | Primer sequence (5’-3’) | Product size (bp) |
| --- | --- | --- |
| TLR4-F | AGCTTTTACCACTATCCAGAGCAA | 157 |
| TLR4-R | GAGAAGGAGGTGGCTTACCC |  |
| MyD88-F | ACCATTCGAGATGACCCCCT | 185 |
| MyD88-R | CTAGCAATGGACCAGACGCA |  |
| NLRP3-F | TTCCATGGCTCAGGACACAC | 85 |
| NLRP3-R | AGCCCTAGTCAGAGTCCCAG |  |
| ASC-F | ACAACAAACCAGCACTGCAC | 123 |
| ASC-R | CCTGGTACTGCTCTTCCGTC |  |
| Caspase-1-F | TACAAGAATCCCAGGCGGTG | 128 |
| Caspase-1-R | CCTTTGGGCTATGTCTGGGG |  |

Table S4. PRRSV isolate TCID50 determination results

| Degree of dilution | Number of cell Wells | Number of holes with CPE | Number of holes without CPE | The cumulative number of CPE holes | Cumulative number of holes without CPE | Total number of cell Wells | Percentage of CPE (%) |
| --- | --- | --- | --- | --- | --- | --- | --- |
| -1 | 8 | 8 | 0 | 47 | 0 | 47 | 100 |
| -2 | 8 | 8 | 0 | 39 | 0 | 39 | 100 |
| -3 | 8 | 8 | 0 | 31 | 0 | 31 | 100 |
| -4 | 8 | 8 | 0 | 23 | 0 | 23 | 100 |
| -5 | 8 | 8 | 0 | 15 | 0 | 15 | 100 |
| -6 | 8 | 5 | 3 | 7 | 3 | 10 | 70 |
| -7 | 8 | 2 | 6 | 2 | 9 | 11 | 18.18 |
| -8 | 8 | 0 | 8 | 0 | 17 | 17 | 0 |
| -9 | 8 | 0 | 8 | 0 | 25 | 25 | 0 |


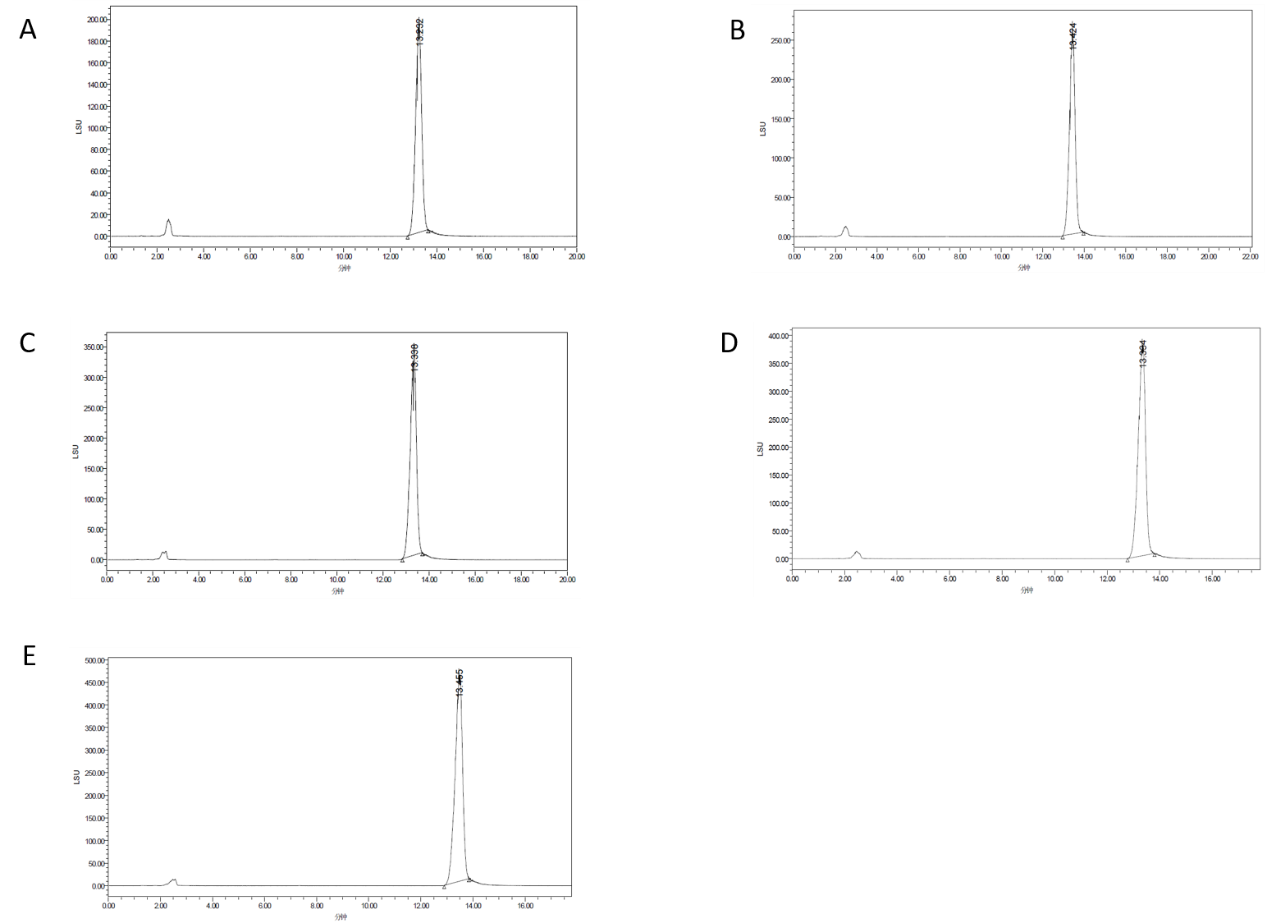


Figure S1 Results of standard determination

A: 8 μl; B: 10 μl; C: 12 μl; D: 14 μl; E: 16 μl
